# A Radiographic Analysis of Coronal Morphological Parameters of Lower Limbs in Chinese Non‐knee Osteoarthritis Populations

**DOI:** 10.1111/os.13952

**Published:** 2023-12-13

**Authors:** Xu Jiang, Kai Xie, Hongyu Chen, Kai Zhang, Yuqi Hu, Tianyou Kan, Lin Sun, Songtao Ai, Xianping Zhu, Lichi Zhang, Mengning Yan, Liao Wang

**Affiliations:** ^1^ Shanghai Frontiers Science Center of Degeneration and Regeneration in Skeletal System, Shanghai Key Laboratory of Orthopaedic Implants, Department of Orthopaedic Surgery Shanghai Jiao Tong University of Medicine affiliated Ninth People's Hospital Shanghai China; ^2^ School of Biomedical Engineering Shanghai Jiao Tong University Shanghai China; ^3^ Department of Radiology Shanghai Jiao Tong University of Medicine affiliated Ninth People's Hospital Shanghai China; ^4^ Department of Orthopaedic Surgery Taizhou Central Hospital Taizhou China

**Keywords:** Artificial Intelligence, Chinese Populations, Knee Osteoarthritis, Lower Limb Morphological Parameters

## Abstract

**Objectives:**

Analyzing the lower limb coronal morphological parameters in populations without knee osteoarthritis (KOA) holds significant value in predicting, diagnosing, and formulating surgical strategies for KOA. This study aimed to comprehensively analyze the variability in these parameters among Chinese non‐KOA populations, employing a substantial sample size.

**Methods:**

A cross‐sectional retrospective analysis was performed on the Chinese non‐KOA populations (n = 407; 49.9% females). The study employed an in‐house developed artificial intelligence software to meticulously assess the coronal morphological parameters of all 814 lower limbs. The parameters evaluated included the hip‐knee‐ankle angle (HKAA), weight‐bearing line ratio (WBLR), joint line convergence angle (JLCA), mechanical lateral‐proximal‐femoral angle (mLPFA), mechanical lateral‐distal‐femoral angle (mLDFA), mechanical medial‐proximal‐tibial angle (mMPTA), and mechanical lateral‐distal‐tibial angle (mLDTA). Differences in these parameters were compared between left and right limbs, different genders, and different age groups (with 50 years as the cut‐off point).

**Results:**

HKAA and JLCA exhibited left–right differences (left vs. right: 178.2° ± 3.0° *vs.* 178.6° ± 2.9° for HKAA, *p* = 0.001; and 1.8° ± 1.5° vs. 1.4° ± 1.6° for JLCA, *p* < 0.001); except for the mLPFA, all other parameters show gender‐related differences (male vs. female: 177.9° ± 2.8° *vs.* 179.0° ± 3.0° for HKAA, *p* < 0.001; 1.5° ± 1.5° vs. 1.8° ± 1.7° for JLCA, *p* = 0.003; 87.1° ± 2.1° vs. 88.1° ± 2.1° for mMPTA, *p* < 0.001; 90.2° ± 4.0° vs. 91.1° ± 3.2° for mLDTA, *p* < 0.001; 38.7% ± 12.9% vs. 43.6% ± 14.1% for WBLR, *p* < 0.001; and 87.7° ± 2.3° vs. 87.4° ± 2.7° for mLDTA, *p* = 0.045); mLPFA increase with age (younger vs. older: 90.1° ± 7.2° *vs.* 93.4° ± 4.9° for mLPFA, *p* < 0.001), while no statistical difference exists for other parameters.

**Conclusions:**

There were differences in lower limb coronal morphological parameters among Chinese non‐KOA populations between left and right sides, different genders, and age.

## Graphical abstract

This study is currently the largest‐scale analysis of lower limb coronal morphological parameters in non‐knee osteoarthritis (KOA) Chinese populations. A total of 407 patients (814 lower limbs) were included according to the inclusion and exclusion criteria. We observed differences in the lower limb coronal morphological parameters between the left and right sides, sex, and age in the non‐KOA Chinese populations.

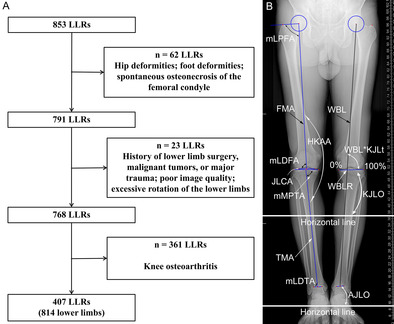

## Introduction

Osteoarthritis (OA) is a significant cause of pain and disability in older populations, with variations in lowerlimb morphological parameters affecting weight‐loading joints and leading to imbalanced mechanical loading, local stress concentration, cartilage wear, and ultimately OA progression.^1^, ^2^, ^3^, ^4^, ^5^, ^6^, ^7^ Studies show that morphological and structural variations in the hip joint closely relate to hip OA development, and morphological characteristics of the pelvis and hip joint relate to different compartment knee OA (KOA).^6^, ^8^ Additionally, local morphological parameters around the knee joint relate to KOA occurrence, and femoral shaft changes with age result in varus deformity of the lower limb, which is closely associated with KOA progression.^9^, ^10^, ^11^ The kinematic alignment in total knee arthroplasty (TKA) supports the concept of restoring the limb to its pre‐disease mechanical axis.^12^, ^13^, ^14^, ^15^, ^16^, ^17^, ^18^, ^19^, ^20^ Many researchers recommended using the contra‐lateral limb as the physiological template.^12^, ^21^, ^22^ Therefore, analyzing the lower limb coronal morphological parameters in non‐KOA populations holds significant value in predicting, diagnosing, and formulating surgical strategies for KOA.

Extensive research has been conducted on the coronal morphological parameters of lower limbs in healthy individuals.^23^, ^24^, ^25^, ^26^, ^27^, ^28^, ^29^, ^30^, ^31^, ^32^, ^33^, ^34^, ^35^, ^36^, ^37^, ^38^ Most studies used radiographic data from the long‐leg anteroposterior radiographs (LLRs); Than et al.^30^ and Hodel et al.^34^ used radiographic data from the EOS imaging, and Hirschmann et al.^35^ and Siboni et al.^36^ used lower limb computed tomography (CT). Although EOS imaging and CT scans can furnish more precise data due to their lower radiation doses and cost‐effectiveness, LLRs remain the preferred choice for large‐scale studies. Only half of the studies had sample sizes >100, with Nakano et al.^38^ having the largest sample size (797 cases) in the Japanese population. There was only one study in Chinese non‐KOA populations with a sample size of 50 cases.^26^ Therefore, there is a dearth of extensive sample studies in Chinese non‐KOA populations. Incorporating individuals of various age groups facilitates the exploration of age‐related trends. However, the majority of studies in non‐KOA populations have encompassed young and middle‐aged individuals, with only Cooke et al.,^25^ Nakano et al.,^38^ Siboni et al.,^36^ and Hodel et al.^34^ involving older individuals. Furthermore, prior studies employed conventional measuring tools, which are time‐consuming and arduous, and results may vary significantly among researchers. The rapid advancements in artificial intelligence (AI) technology have led to the development of automated measurement programs based on AI technology that offers accuracy comparable to that of experts and efficiency surpassing human capabilities, thus becoming reliable tools for big data epidemiological research.^39^, ^40^, ^41^, ^42^, ^43^

Therefore, this study used AI technology to comprehensively analyze the variability in lower limb coronal morphological parameters in Chinese non‐KOA populations with a substantial sample size. The overarching objectives encompass the elucidation of disparities between (i) the left and right anatomical aspects, (ii) the gender‐based distinctions, and (iii) the variations across various age strata.

## Methods

### Study Population

**FIGURE 1:**
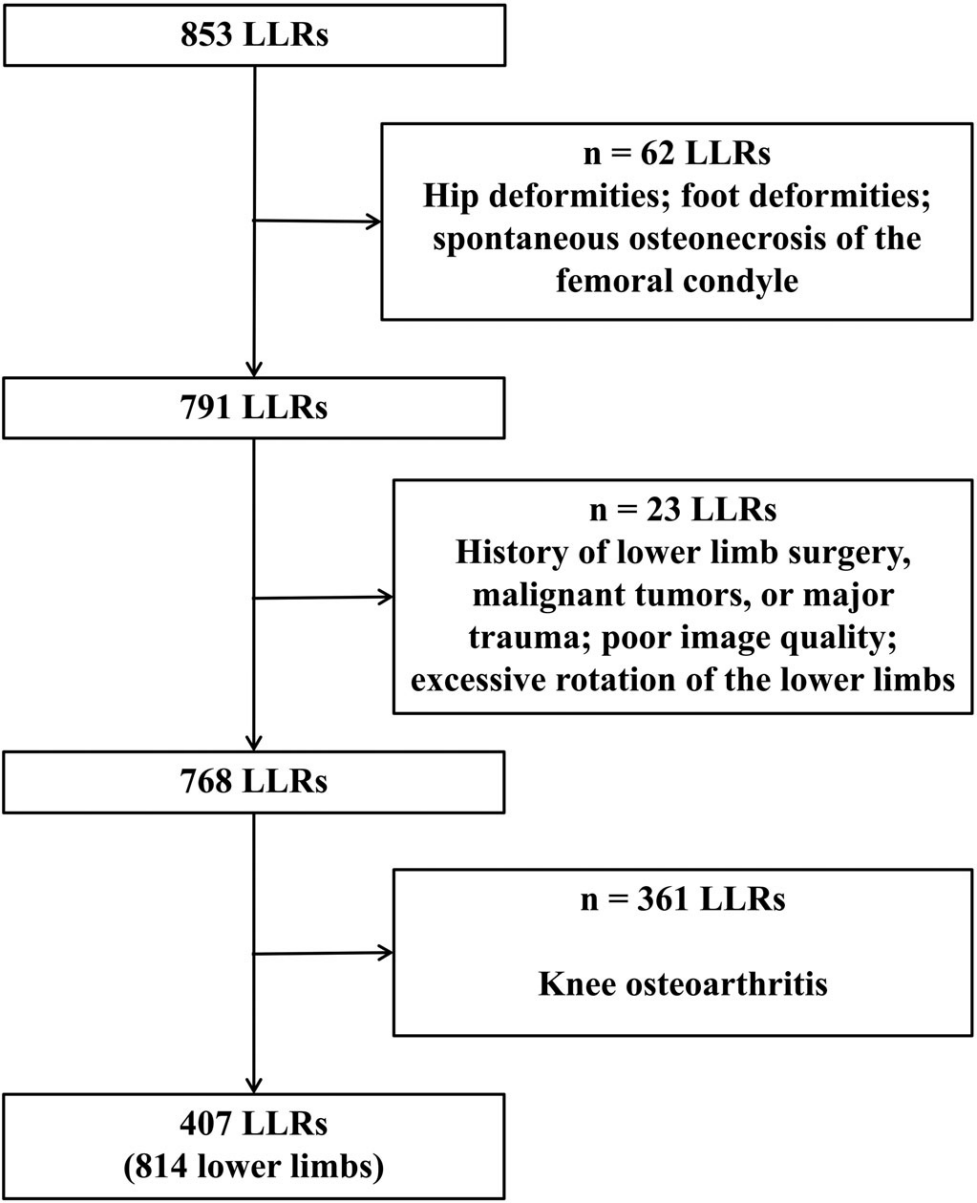
Flowchart of participant selection.

This cross‐sectional retrospective study was approved by our IRB (No. SH9H‐2023‐T97‐1). We collected LLRs of both lower limbs using a Siemens Medical cone‐beam computed tomography (CBCT) DR machine (Multitom Rax, Siemens Medical, Erlangen, Germany) at our hospital from December 2020 to October 2022. The inclusion criteria were adult patients with the earliest available LLR. The exclusion criteria were as follows: (1) developmental dysplasia of the hip, hip OA, femoral head necrosis, and other hip deformities; (2) foot deformities such as clubfoot deformity; (3) spontaneous osteonecrosis of the femoral condyle; (4) history of lower limb surgery, malignant tumors, or major trauma; (5) poor image quality; (6) excessive rotation of the lower limbs; and (7) KOA. Ultimately, we included 407 non‐KOA populations (814 lower limbs) with a mean age of 44.5 ± 17.2 years, ranging from 18 to 85 years: 204 males with a mean age of 44.5 ± 17.4 years, ranging from 18 to 85 years; and 203 females with a mean age of 44.4 ± 16.9 years, ranging from 18 to 84 years. The selection process for the image data is illustrated in Figure 1.

### Definition and Measurement of Morphological Parameters

**FIGURE 2:**
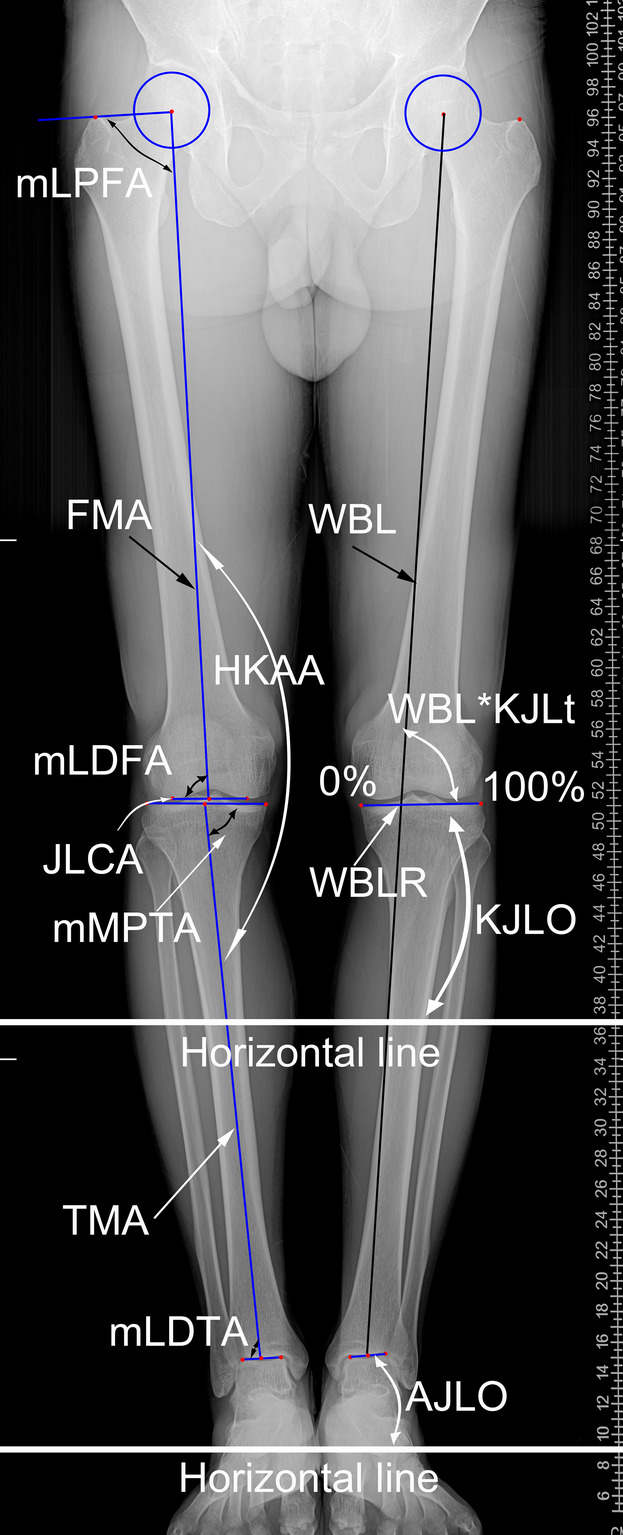
Definition of anatomical landmarks and parameters. FMA, femoral mechanical axis; WBL, weight‐bearing line; TMA, tibial mechanical axis; KJLt, knee joint line of tibial side; mLPFA, mechanical lateral‐proximal‐femoral angle; mLDFA, mechanical lateral‐distal‐femoral angle; JLCA, joint line convergence angle; mMPTA, mechanical medial‐proximal‐tibial angle; mLDTA, mechanical lateral‐distal‐tibial angle; HKAA, hip‐knee‐ankle angle; WBLR, weight‐bearing line ratio; KJLO, knee joint line orientation; AJLO, ankle joint line orientation.

The lower limb coronal morphological parameters, including hip‐knee‐ankle angle (HKAA), weight‐bearing line ratio (WBLR), joint line convergence angle (JLCA), mechanical lateral‐proximal‐femoral angle (mLPFA), mechanical lateral‐distal‐femoral angle (mLDFA), mechanical medial‐proximal‐tibial angle (mMPTA), and mechanical lateral‐distal‐tibial angle (mLDTA), were determined as described in previous studies (Figure 2).^44^, ^45^

We carefully analyzed the definitions of different anatomical parameters in previous studies and found that in some studies, authors use the mechanical medial‐distal‐femoral angle (mMDFA) and mechanical lateral‐proximal‐tibial angle (mLPTA) instead of mLDFA and mMPTA. It's noteworthy that mMDFA and mLPTA are complementary angles to mLDFA and mMPTA. To facilitate comparison with other studies, we converted mMDFA and mLPTA into mLDFA and mMPTA. All lower limb morphological parameters of the LLRs were evaluated using an in‐house developed AI software (https://skyw.ltd/).

### Grouping

All non‐KOA populations with both lower limbs were included in the study and divided into left and right groups. The patients were divided into male and female groups according to gender. Patients were also divided into younger (<50 years) and older (≥50 years) groups based on age, and further subgroups were classified based on gender in both the younger and older groups.

### Statistical Analysis

Descriptive statistics were reported as means ± standard deviation (SD), 95% confidence intervals (CI), and percentages. Independent sample *t*‐tests were used for comparisons between different genders and different age groups. Paired sample *t*‐tests were used for comparisons between the left and right lower limbs, and summary‐independent sample *t*‐tests were used for comparisons with previous studies. All statistical analyses were conducted using SPSS v23 software (IBM Corp., New York, USA), and a *p*‐value <0.05 was considered statistically significant.

## Results

### Comparison between Left and Right Lower Limbs

Table 1 shows the results of the comparison of the morphological parameters between the left and right lower limbs. Except for HKAA, JLCA, and WBLR, which showed significant differences between the left and right sides (left vs. right: 178.2° ± 3.0° vs. 178.6° ± 2.9° for HKAA, *p* = 0.001; 1.8° ± 1.5° vs. 1.4° ± 1.6° for JLCA, *p* < 0.001; and 40.2% ± 13.8% vs. 42.1% ± 13.7% for WBLR, *p* = 0.001), there were no significant differences in the other parameters.

**TABLE 1:** Comparison of morphological parameters between the left and right lower limbs.

Parameters	Left (n = 407)	Right (n = 407)	*p*‐value	Overall (n = 814)
HKAA (°)	178.2 ± 3.0 (177.9–178.5)	178.6 ± 2.9 (178.3–178.9)	0.001	178.4 ± 3.0 (178.2–178.6)
mLDFA (°)	87.6 ± 2.4 (87.3–87.8)	87.6 ± 2.6 (87.3–87.8)	0.965	87.6 ± 2.5 (87.4–87.7)
JLCA (°)	1.8 ± 1.5 (1.7–2.0)	1.4 ± 1.6 (1.3–1.6)	<0.001	1.6 ± 1.6 (1.5–1.7)
mMPTA (°)	87.6 ± 2.2 (87.4–87.8)	87.6 ± 2.2 (87.4–87.8)	0.823	87.6 ± 2.2 (87.5–87.8)
mLPFA (°)	91.1 ± 5.6 (90.6–91.7)	91.7 ± 7.4 (91.0–92.4)	0.076	91.4 ± 6.6 (91.0–91.9)
mLDTA (°)	90.7 ± 4.0 (90.3–91.1)	90.6 ± 3.3 (90.3–91.0)	0.682	90.7 ± 3.7 (90.4–90.9)
WBLR (%)	40.2 ± 13.8 (38.9–41.6)	42.1 ± 13.7 (40.8–43.4)	0.001	41.2 ± 13.8 (40.2–42.1)

Abbreviations: HKAA, hip‐knee‐ankle angle; JLCA, joint line convergence angle; mLDFA, mechanical lateral‐distal‐femoral angle; mLDTA, mechanical lateral‐distal‐tibial angle; mLPFA, mechanical lateral‐proximal‐femoral angle; mMPTA, mechanical medial‐proximal‐tibial angle; WBLR, weight‐bearing line ratio.

### Comparison among Gender‐Related Groups

Table 2 shows a comparison of lower limb morphological parameters among the gender‐related group. The male group comprised 204 patients (408 lower limbs), and the female group consisted of 203 patients (406 lower limbs), with no significant difference in age between the two groups. Except for mLPFA, all other morphological parameters showed significant differences among the gender‐related groups. Males had smaller values for HKAA, JLCA, mMPTA, mLDTA, and WBLR and larger values for mLDFA than females (male vs. female: 177.9° ± 2.8° vs. 179.0° ± 3.0° for HKAA, *p* < 0.001; 1.5° ± 1.5° vs. 1.8° ± 1.7° for JLCA, *p* = 0.003; 87.1° ± 2.1° vs. 88.1° ± 2.1° for mMPTA, *p* < 0.001; 90.2° ± 4.0° vs. 91.1° ± 3.2° for mLDTA, *p* < 0.001; 38.7% ± 12.9% vs. 43.6% ± 14.1% for WBLR, *p* < 0.001; and 87.7° ± 2.3° vs. 87.4° ± 2.7° for mLDFA, *p* = 0.045).

**TABLE 2:** Comparison of lower limb morphological parameters between genders.

Parameters	Male (n = 204)	Female (n = 203)	*p*‐value
Age (years)	44.5 ± 17.4 (42.8–46.2)	44.4 ± 16.9 (42.7–46.0)	0.904
HKAA (°)	177.9 ± 2.8 (177.6–178.2)	179.0 ± 3.0 (178.7–179.3)	<0.001
mLDFA (°)	87.7 ± 2.3 (87.5–88.0)	87.4 ± 2.7 (87.1–87.6)	0.045
JLCA (°)	1.5 ± 1.5 (1.3–1.6)	1.8 ± 1.7 (1.6–2.0)	0.003
mMPTA (°)	87.1 ± 2.1 (86.9–87.3)	88.1 ± 2.1 (87.9–88.3)	<0.001
mLPFA (°)	91.7 ± 6.2 (91.1–92.3)	91.1 ± 6.9 (90.4–91.8)	0.209
mLDTA (°)	90.2 ± 4.0 (89.8–90.6)	91.1 ± 3.2 (90.8–91.4)	<0.001
WBLR (%)	38.7 ± 12.9 (37.5 – −40.0)	43.6 ± 14.1 (42.2–45.0)	<0.001

Abbreviations: HKAA, hip‐knee‐ankle angle; mLDFA, mechanical lateral‐distal‐femoral angle; JLCA, joint line convergence angle; mMPTA, mechanical medial‐proximal‐tibial angle; mLPFA, mechanical lateral‐proximal‐femoral angle; mLDTA, mechanical lateral‐distal‐tibial angle; WBLR, weight‐bearing line ratio.

### Comparison between Younger and Older Groups

Table 3 shows the results of the comparison of the lower limb morphological parameters between the younger and older groups. There were 246 and 161 patients (492 and 322 lower limbs) in the younger and older groups, respectively. The comparison between the two groups showed that mLPFA increased with age (younger vs. older: 90.1° ± 7.2° vs. 93.4° ± 4.9° for mLPFA, *p* < 0.001), while the remaining parameters showed no statistically significant differences.

**TABLE 3:** Comparison of lower limb morphological parameters between the younger and older groups.

Parameters	Younger group (<50 years, n = 246)	Older group (≥50 years, n = 161)	*p*‐value
HKAA (°)	178.4 ± 2.9 (178.1–178.6)	178.5 ± 3.0 (178.1–178.8)	0.717
mLDFA (°)	87.6 ± 2.4 (87.4–87.9)	87.4 ± 2.5 (87.2–87.7)	0.241
JLCA (°)	1.6 ± 1.6 (1.5–1.7)	1.7 ± 1.5 (1.5–1.8)	0.576
mMPTA (°)	87.6 ± 2.2 (87.4–87.8)	87.6 ± 2.1 (87.3–87.8)	0.649
mLPFA (°)	90.1 ± 7.2 (89.4–90.7)	93.4 ± 4.9 (92.9–94.0)	<0.001
mLDTA (°)	90.7 ± 3.8 (90.3–91.0)	90.7 ± 3.5 (90.3–91.1)	0.939
WBLR (%)	40.6 ± 13.8 (39.4–41.9)	41.9 ± 13.6 (40.4–43.4)	0.193

Abbreviations: HKAA, hip‐knee‐ankle angle; JLCA, joint line convergence angle; mLDFA, mechanical lateral‐distal‐femoral angle; mLDTA, mechanical lateral‐distal‐tibial angle; mLPFA, mechanical lateral‐proximal‐femoral angle; mMPTA, mechanical medial‐proximal‐tibial angle; WBLR, weight‐bearing line ratio.

### Subgroups Analysis

We compared the differences among different genders in the younger and older age groups and age‐related changes in morphological parameters between the male and female subgroups, as shown in Table 4. In the younger group, there were 128 male and 118 female patients (256 and 236 lower limbs), while in the older group, there were 76 male and 85 female patients (152 and 85 lower limbs). Males in the younger group had smaller HKAA, JLCA, mMPTA, and WBLR, and larger mLDFA and mLPFA than females, with no statistical differences in the remaining parameters. Moreover, males in the older group had lower HKAA, JLCA, mMPTA, mLDTA, and WBLR than females, with no statistical differences in the remaining parameters. A comparison of parameters between male and female subgroups in the younger and older groups showed that mLPFA in gender‐related subgroups increased with age, whereas mLDFA in male subgroup decreased with age. There were no statistical differences in the remaining parameters.

**TABLE 4:** Differences between genders in the younger and older groups.

Parameters	Younger group (<50 years)			Older group (≥50 years)			*p*‐value (Male)	*p*‐value (Female)
	Male (n = 128)	Female (n = 118)	*p*‐value	Male (n = 76)	Female (n = 85)	*p*‐value		
HKAA (°)	177.8 ± 2.9 (177.5–178.2)	179.0 ± 2.9 (178.6–179.4)	<0.001	178.0 ± 2.7 (177.5–178.4)	178.9 ± 3.1 (178.4–179.4)	0.005	0.604	0.758
mLDFA (°)	87.9 ± 2.4 (87.6–88.2)	87.4 ± 2.5 (87.0–87.7)	0.012	87.4 ± 2.1 (87.1–87.8)	87.4 ± 2.9 (87.0–87.9)	0.964	0.039	0.793
JLCA (°)	1.5 ± 1.5 (1.3–1.6)	1.7 ± 1.7 (1.5–2.0)	0.050	1.5 ± 1.4 (1.2–1.7)	1.8 ± 1.7 (1.6–2.1)	0.025	1.000	0.584
mMPTA (°)	87.2 ± 2.2 (86.9–87.5)	88.1 ± 2.1 (87.8–88.4)	<0.001	86.9 ± 2.1 (86.5–87.2)	88.2 ± 2.0 (87.9–88.5)	<0.001	0.136	0.728
mLPFA (°)	90.8 ± 6.8 (90.0–91.6)	89.3 ± 7.4 (88.3–90.2)	0.019	93.2 ± 4.7 (92.4–94.0)	93.7 ± 5.1 (92.9–94.4)	0.406	<0.001	<0.001
mLDTA (°)	90.4 ± 4.3 (89.8–90.9)	91.0 ± 3.2 (90.6–91.4)	0.066	90.0 ± 3.6 (89.4–90.6)	91.3 ± 3.3	0.001	0.346	0.339
WBLR (%)	38.2 ± 13.4 (36.6–39.9)	43.3 ± 13.8 (41.5–45.1)	<0.001	39.6 ± 12.1 (37.7–41.6)	44.0 ± 14.5 (41.8–46.2)	0.003	0.280	0.621

Abbreviations: HKAA, hip‐knee‐ankle angle; JLCA, joint line convergence angle; mLDFA, mechanical lateral‐distal‐femoral angle; mLDTA, mechanical lateral‐distal‐tibial angle; mLPFA, mechanical lateral‐proximal‐femoral angle; mMPTA, mechanical medial‐proximal‐tibial angle; WBLR, weight‐bearing line ratio.

### Comparison with Other Studies

Table 5 presents demographic data from previous studies on lower limb morphological parameters in the non‐KOA population. In Table 6, we compared the differences in HKAA, mLDFA, mMPTA, and JLCA between previous studies and the results of this study. Among the 16 relevant studies, eight had a sample size of fewer than 100 cases and were thus excluded. In Caucasian studies, the differences between the Canadian population of Cooke et al.^25^ and our study population were mainly reflected in smaller mLDFA and mMPTA; the European American population of Siboni et al.^36^ had smaller mLDFA, mMPTA, and JLCA than those in our study population. The Belgian population of Bellemanns et al.^29^ differed from our study population in mLDFA, mMPTA, and JLCA, and mLDFA was slightly larger than that of our results, as well as mMPTA and JLCA, which were smaller than that our results. Because Hirschmann et al.^35^ and Siboni et al.^36^ used CT data, both of their HKAA were biased toward a neutral position. In Asian studies, the male subgroup in the Iranian population of Jabalameli et al.^33^ had smaller HKAA, mMPTA, and JLCA than those in our study population; the Indian/Korean population of Shetty et al.^31^ had smaller HKAA and mMPTA than those of our study population. Although the Korean population of Song et al.^32^ had no difference in HKAA from those in our study population, the female subgroup had larger mLDFA and smaller mMPTA than those in our study population. Both the male and female subgroups in the Japanese population of Nakano et al.^38^ had a smaller mMPTA than those in our study population.

**TABLE 5:** Demographic data from previous studies on lower limb morphological parameters in the non‐KOA population.

Authors	Published years	Scanner	Sample size (Male/Female)	Country	Age, years (Mean ± SD, /range)
Moreland^23^	1987	LLR	25 (25/0)	USA	30, 25–45
Hsu^24^	1990	LLR	60 (30/30)	USA	–
Cooke^25^	1997	LLR	119 (52/67)	Canada	24 ± 3 (younger group)
					66 ± 8 (older group)
Tang^26^	2000	LLR	50 (25/25)	China	24, 22–31 (Male)
					23, 21–29 (Female)
Mullaji^27^	2009	LLR	25 (25/0)	India	32, 21–39 (Male)
Khattak^28^	2010	LLR	59 (40/19)	Pakistan	20–45
Bellemanns^29^	2011	LLR	250 (125/125)	Belgium	20–27
Than^30^	2012	EOS imaging	65 (29/36)	Hungary	26.3, 19–39
Shetty^31^	2014	LLR	194 (102/92)	India/Korea	20–40
Song^32^	2015	LLR	118 (0/118)	Korea	26.8 ± 4.3 (Female)
Jabalameli^33^	2015	LLR	100 (50/50)	Iran	24.4 ± 3.8 (Male)
					25.5 ± 4.1 (Female)
Nakano^38^	2016	LLR	797 (272/525)	Japan	17–88
Tanaka^37^	2017	LLR	34 (12/22)	Japan	26.4 ± 7.8
Hirschmann^35^	2019	CT	160 (102/58)	Switzerland	30 ± 7, 16–44
Siboni^36^	2021	CT	586 (269/317)	USA	61.9 ± 14.8, 21–92
Hodel^34^	2022	EOS imaging	30 (14/16)	Switzerland	27.1 ± 10, 20–67
Our study	2023	LLR	407 (204/203)	China	44.5 ± 17.2, 18–85

Abbreviations: HKAA, hip‐knee‐ankle angle; JLCA, joint line convergence angle; LLRs, long‐leg anteroposterior radiographs; mLDFA, mechanical lateral‐distal‐femoral angle; mLDTA, mechanical lateral‐distal‐tibial angle; mLPFA, mechanical lateral‐proximal‐femoral angle; mMPTA, mechanical medial‐proximal‐tibial angle; SD, standard deviation; WBLR, weight‐bearing line ratio.

**TABLE 6:** Differences in HKAA, mLDFA, mMPTA, and JLCA between previous studies and this study.

Authors	HKAA (n, °)			mLDFA (n, °)			mMPTA (n, °)			JLCA (n, °)		
	Male	Female	Overall	Male	Female	Overall	Male	Female	Overall	Male	Female	Overall
Moreland^23^	178.5 ± 2.0 (25, right)						87.0 ± 1.6 (25, right)					
	178.9 ± 2.0 (25, left)						87.4 ± 1.4 (25, left)					
Hsu^24^			178.8 ± 2.2 (120)									
Cooke^25^	178.4 ± 2.8 (52)	179.5 ± 2.8 (67)	179.0 ± 2.9 (119)*	86.5 ± 2.2 (52)***	85.7 ± 2.4 (67)***	86.0 ± 2.3 (119)***	86.6 ± 2.3 (52)	87.2 ± 2.3 (67)***	86.9 ± 2.3 (119)***	1.7 ± 1.5 (52)	2.0 ± 1.7 (67)	1.9 ± 1.6 (119)
Tang^26^	177.8 ± 2.7 (50)	177.8 ± 2.5 (50)**					85.1 ± 2.3 (50)***	84.6 ± 2.5 (50)***				
Mullaji^27^	178.7 ± 0.8 (50)*			86.9 ± 1.6 (50)*								
Khattak^28^	178.4 ± 2.8 (80)	180.0 ± 3.0 (38)					86.6 ± 2.2 (80)	88.6 ± 3.2 (38)				
Bellemanns^29^	178.1 ± 2.4 (250)	179.2 ± 2.1 (250)	178.7 ± 2.3 (500)	87.9 ± 1.7 (250)	87.9 ± 1.8, (250)*	87.9 ± 1.7 (500)*	86.5 ± 2.2 (250)***	87.6 ± 1.8 (250)**	87.0 ± 2.1 (500)***	0.5 ± 1.0 (250)***	0.6 ± 1.1 (250)***	0.5 ± 1.1 (500)***
Than^30^			179.2 ± 3.1 (128)**						88.0 ± 3.6 (128)			
Shetty^31^	177.2 ± 2.5 (184)**	178.0 ± 2.5 (204)***	177.6 ± 2.6 (388)***						86.7 ± 1.9 (388)***			
Song^32^		178.7 ± 2.0 (236)			87.8 ± 1.7 (236)*			86.8 ± 1.6 (236)***				
Jabalameli^33^	177.0 ± 3.1 (100)**	179.3 ± 2.7 (100)	178.5 ± 2.9 (200)				86.4 ± 1.7 (100)**	88.0 ± 2.0 (100)	87.2 ± 2.0 (200)*	1.0 ± 1.4 (100)**	1.0 ± 1.7 (100)***	1.0 ± 1.6 (200)***
Nakano^38^							85.6 ± 2.2 (797)***	85.1 ± 2.4 (797)***				
Tanaka^37^			179.7 ± 2.0 (34)*									
Hirschmann^35^	179.2 ± 2.8 (195)***	180.5 ± 2.8 (113)***	179.5 ± 2.9 (308)***									
Siboni^36^			180.0 ± 2.6 (586)***			86.1 ± 1.9 (586)***			86.1 ± 2.2 (586)***			0.8 ± 1.3 (586)***
Hodel^34^			178.3 ± 3.7 (60)									
Our study	177.9 ± 2.8 (408)	179.0 ± 3.0 (406)	178.4 ± 3.0 (814)	87.7 ± 2.3 (408)	87.4 ± 2.7 (406)	87.6 ± 2.5 (814)	87.1 ± 2.1 (408)	88.1 ± 2.1 (406)	87.6 ± 2.2 (814)	1.5 ± 1.5 (408)	1.8 ± 1.7 (406)	1.6 ± 1.6 (814)

Abbreviation: n, the number of lower limbs.

* *p* < 0.05.

** *p* < 0.01.

*** *p* < 0.001.

## Discussion

This study is currently the largest‐scale analysis of lower limb coronal morphological parameters in the non‐KOA Chinese population. We observed differences in the lower limb coronal morphological parameters between the left and right sides, different genders, and different age groups in the non‐KOA Chinese populations, which holds significant value in predicting, diagnosing, and formulating surgical strategies for KOA.

### Left–Right Differences

This study found left–right differences in lower limb coronal morphology parameters in non‐KOA Chinese populations. The overall morphological parameters that differed between the left and right sides were HKAA and WBLR, whereas the local morphological parameter that differed was JLCA, which was more varus on the left than on the right side. Because HKAA = mMPTA + (180° − mLDFA) − JLCA, and mMPTA and mLDFA do not differ between the left and right sides, the overall morphological differences between the left and right sides are mainly due to the difference in JLCA, which is larger on the left side. The kinematic alignment in total knee arthroplasty (TKA) supports the concept of restoring the limb to its pre‐disease mechanical axis, accepting an outcome that deviates from the target neutral mechanical axis target.^12^, ^13^, ^14^, ^15^, ^16^, ^17^, ^18^, ^19^, ^20^ Many researchers recommended using the contra‐lateral limb as the physiological template.^12^, ^21^, ^22^ Therefore, analyzing the morphological parameters of the left and right lower limbs can provide valuable information to guide the planning and execution of anatomical reconstruction in TKA.

Dargel et al. analyzed 71 morphological features in a cadaveric study and found differences only in the posterior slope of the tibial plateau, anatomical valgus of the distal femur, and position of the anterior cruciate ligament insertion.^46^ Moreover, Eckhof et al. and Jacquet et al. used CT data and found that coronal morphological parameters were symmetrical between the left and right sides.^47^, ^48^ Eckstein et al. used MRI data to analyze the cartilage thickness in the left and right femurs and suggested using the contralateral femur as a reference.^49^ Because CT/MRI and cadaveric studies are performed in non‐weight‐bearing positions, they cannot reflect the weight‐bearing status of the JLCA and HKAA. Therefore, using the unaffected contralateral limb as a physiological template for correcting knee‐joint alignment during TKA is questionable. Based on Bellemann et al.'s^29^ LLRs data, Beckers et al.^50^ showed that only 79% of the lower limb HKAA were in the same varus‐valgus group, and when considering mLDFA and mMPTA, only 59% of the lower limb morphological parameters were in the same group. Although there were no statistically significant differences between the left and right sides, the correlation between the two sides was mostly weak, and only 60% of the left HKAA was within ±3° of the right HKAA range. Therefore, Beckers et al. believed that the evidence for using a healthy lower limb as a physiological template for TKA correction was insufficient. Our results support the asymmetry hypothesis proposed by Beckers et al.,^50^ and we found that this asymmetry originates from the asymmetry of the JLCA. In non‐KOA populations, JLCA changes may be caused by loosening of the medial or lateral collateral ligaments or abnormalities in other parts.^51^ Although the human body is initially symmetrical, various factors in growth and life can cause abnormal coronal plane posture, which ultimately changes the load distribution of the lower limbs during standing and thus alters the symmetry of the JLCA. This alteration may potentially lead to the development of knee OA. In addition, pain experienced by patients with OA may cause them to rely more on their unaffected limbs, which can also cause changes in the JLCA and HKAA.^52^

### Gender‐Related Differences

In this study, except for mLPFA, all parameters showed gender‐related differences. Compared with females, males had varus HKAA, mLDFA, and mMPTA, as well as valgus JLCA. The gender‐related difference in mMPTA was much greater than that in mLDFA and JLCA. Therefore, the main reason for the greater varus HKAA in males was that the varus mMPTA was also greater. In clinical practice, male patients are more likely to undergo high tibial osteotomies, and the results of this study support this phenomenon. Smaller mLDTA in males reflects HKAA affecting the ankle joint, and the varus knee may lead to a decrease in mLDTA, which is consistent with the conclusions of Xie et al.^5^

Almost all studies support greater varus HKAA in males; however, there is a controversy about whether gender‐related differences exist in local parameters. The differences in HKAA and mMPTA between males and females in the studies by Khattak et al.^28^ and Jabalameli et al.^33^ were consistent with the results of this study; however, there was no significant difference in JLCA between males and females by Jabalameli et al.^33^ Bellemanns et al.^29^ found no significant difference in mLDFA and JLCA between males and females, supporting the suggestion that the main source of greater varus HKAA in males is greater varus mMPTA. However, Cooke et al.^25^ believed that the main reason for the greater varus HKAA in males was the more varus mLDFA, and the parameter difference between males and females for mLDFA was greater than that for mMPTA and JLCA. These differences may be attributed to the heterogeneity of the study population in terms of race, age, and inclusion/exclusion criteria for participants.

### Age Differences

This study found that except for mLPFA, all other parameters showed no significant differences with increasing age. mLPFA was determined using the midpoint of the KJLf, which was the knee joint center of the femoral side (KJLf midpoint), the tip of the greater trochanter (TGR), and the hip joint center (HJC). Because the distance between the KJLf midpoint and TGR was relatively large, its influence on the mLPFA was relatively small. Therefore, the increase in mLPFA may be due to the relatively higher position of the TGR or the relatively lower position of the HJC. Because when the TGR is subjected to little stress, it is unlikely to undergo deformation, and it is more likely that the position of the HJC will decrease. There are two possible reasons for the relative decrease in the HJC position, as follows: (1) deformation of the femoral head or (2) enlargement of the lateral bending of the femur. Matsumoto et al. suggested that the femur changes from medial to lateral bending with increasing age, and the neck‐shaft angle decreases. Although the neck‐shaft angle and mLPFA are local parameters of the hip joint, their changes are opposite. Therefore, our findings are consistent with those of the previous study.

### Subgroups Analysis

According to subgroups analysis, the gender‐related difference in mMPTA was greater than that in mLDFA and JLCA, the mLDFA showed no gender‐related difference in the older group, and the gender‐related difference in the mMPTA was greater than that in the JLCA. These findings support the conclusion that the primary reason for the greater varus HKAA in males was the greater varus mMPTA. We also found that mLPFA in gender‐related subgroups increased with age, which supports the findings of age differences. We observed an intriguing phenomenon: the mLDFA in the female subgroup remained relatively stable with age, while in males, the mLDFA decreased with age until it exhibited no significant difference compared to females. Further research is needed to explore the underlying reasons for this trend.

### Differences among Different Study Populations

Our definitions of mMPTA and JLCA differ from those used in other studies. According to the previous results, our mMPTA and JLCA measurements were approximately 0.5° larger than those obtained using traditional methods. Therefore, the differences between our results and those of Bellemanns et al.^29^ and Cooke et al.^25^ for mMPTA are relatively small. The definitions of mMPTA used by Shetty et al.^31^ and Jabalameli et al.^33^ differ significantly from ours. Therefore, the differences in mMPTA are not meaningful. The differences among races may be due to multiple factors, including genetics, environment, and lifestyle, and further research is required. The normal values for traditional mMPTA and mLDFA were determined based on Caucasian populations, and Paley et al. believed that the ideal values for mMPTA were 87° and 88° for mLDFA.^53^, ^54^ Our study determined the normal values for mMPTA and mLDFA in the Chinese population. In the male, the mLDFA value was larger (87.7° ± 2.3° *vs*. 87.4° ± 2.7°) and mMPTA was smaller (87.1° ± 2.1° *vs*. 88.1° ± 2.1°) than the overall normal value of approximately 87.6°. The difference in mMPTA among the gender‐related groups was greater than in other groups. Therefore, it is necessary to establish Chinese lower limb morphological parameter abnormality standards by gender.

### Limitations and Strengths

Our study has some limitations. First, our participants were recruited from hospital databases rather than normal volunteers. Though the inclusion and exclusion criteria were strict, it is still possible that patients with underlying lower limb diseases were included. Second, lower limb rotation can influence the morphological parameters of the lower limbs. Although we standardized the position of LLR imaging and excluded patients with excessive rotation, we could not eliminate the influence of rotation on the measurement results. Thirdly, in addition to lower limb diseases, spinal deformity, and certain other neuromuscular disorders can also affect coronal morphological parameters. We did not exclude patients with these conditions. Finally, differences in loading between the two lower limbs can cause changes in the JLCA, leading to differences in the bilateral HKAA. Meanwhile, our study could not exclude patients with different loads on the lower limbs.

Our study offers several advantages. First, it represents the largest‐scale investigation of lower limb coronal morphological parameters within the non‐KOA Chinese population, making a substantial contribution to existing knowledge. Second, our study sheds light on left–right asymmetry, gender‐related differences, and age‐related trends in lower limb morphology, offering valuable insights that can inform treatment approaches and surgical decisions. Finally, by establishing Chinese‐specific normative values for morphological parameters categorized by gender, we address a literature gap and provide practical guidance for orthopaedic practice.

## Conclusion

There are differences in lower limb coronal morphological parameters among non‐KOA Chinese populations, including left–right, gender‐related, and age differences. Left–right differences in Chinese populations are mainly due to differences in JLCA. Moreover, this study showed that the greater varus mMPTA contributes to a greater varus HKAA in Chinese males, and the mLPFA increases with age. These findings have important implications for clinical practice and may inform the development of personalized patient treatment plans.

## Disclosure Statement

All authors declare that they have no conflicts of interest.

## Ethical Statement

All procedures performed in studies involving human participants were in accordance with the ethical standards of the institutional and national research committee and with the 1964 Helsinki Declaration and its later amendments or comparable ethical standards. This retrospective study was conducted with the approval of the institutional ethics committee of Shanghai Jiao Tong University of Medicine affiliated Ninth People's Hospital (IRB No. SH9H‐2023‐T97‐1).

## Authors' Contributions

All authors had full access to the data in the study and take responsibility for the integrity of the data and the accuracy of the data analysis. Study concept and design: X.J., K.X., H.Y.C., Y.Q.H., L.C.Z., M.N.Y., and L.W. Acquisition of data: X.J., H.Y.C., K.Z., T.Y.K., L.S., and S.T.A. Analysis and interpretation of the data: X.J., K.X., H.Y.C., K.Z., T.Y.K., X.P.Z., M.N.Y., and L.W. Drafting of the manuscript: X.J., K.X., and H.Y.C. Critical revision of the manuscript for important intellectual content: K.Z., Y.Q.H., T.Y.K., L.S., S.T.A., X.P.Z., L.C.Z., M.N.Y., and L.W. Statistical analysis: X.J. and K.X. Obtained funding: K.X., M.N.Y., and L.W. Administrative, technical and material support: M.N.Y. and L.C.Z. Study supervision: L.W.

## Consent to Participate and Consent for Publication

Due to the retrospective nature of our study, written informed consent was exempted under IRB approval.

## Code Availability (Software Application or Custom Code)

The software application is open source, and we can provide the software application used in the current study if necessary. The web version of the software is at https://skyw.ltd/.

## Authorship Declaration

All authors listed meet the authorship criteria according to the latest guidelines of the International Committee of Medical Journal Editors, and all authors are in agreement with the manuscript.

## Supporting information


**Data S1.** Supporting Information.

## Data Availability

Transparency of the data could be provided, if necessary.
