# Supplementary material for: A Radiographic Analysis of Coronal Morphological Parameters of Lower Limbs in Chinese Non‐knee Osteoarthritis Populations
Source: Orthop Surg. 2023 Dec 13;16(2):452–61. doi: 10.1111/os.13952 (PMC10834221; doi:10.1111/os.13952)
Supplement: Supplementary file 1 — Data S1. Supporting Information. [file OS-16-452-s001.docx]

**Supplementary materials**

1. **Tables**
2. **Background**
3. **Methods**
4. **Results**
5. **References**
6. **Tables**

Table 1S The intra-class correlation coefficient on the testing dataset^a^

| Anatomical parameters | SS-A vs SS-B | JR-A vs JR-B | JR-A vs SS-M | AI vs SS-M |
| --- | --- | --- | --- | --- |
|  | ICC (95% CI) | | | |
| HKAA | 0.998 (0.998-0.999) | 0.998 (0.998-0.999) | 0.997 (0.996-0.998) | 0.999 (0.998-0.999) |
| mLDFA | 0.976 (0.965-0.983) | 0.905 (0.867-0.933) | 0.914 (0.857-0.946) | 0.965 (0.951-0.976) |
| mLPFA | 0.937 (0.911-0.956) | 0.942 (0.918-0.959) | 0.943 (0.918-0.960) | 0.950 (0.918-0.968) |
| mLDTA | 0.963 (0.947-0.974) | 0.976 (0.966-0.983) | 0.980 (0.971-0.986) | 0.958 (0.940-0.970) |
| WBLR | 0.997 (0.996-0.998) | 0.996 (0.993-0.998) | 0.996 (0.991-0.998) | 0.998 (0.997-0.999) |
| AJLO | 0.989 (0.985-0.993) | 0.982 (0.974-0.987) | 0.988 (0.984-0.992) | 0.970 (0.958-0.979) |
| FMA/TMA | 0.989 (0.985-0.993) | 0.993 (0.990-0.995) | 0.991 (0.987-0.994) | 0.991 (0.987-0.994) |
| mMPTA1 | 0.946 (0.923-0.962) | 0.919 (0.886-0.942) | 0.891 (0.848-0.923) | 0.941 (0.917-0.959) |
| mMPTA2 | 0.957 (0.940-0.970) | 0.954 (0.934-0.967) | 0.967 (0.954-0.977) | 0.976 (0.965-0.983) |
| JLCA1 | 0.940 (0.915-0.958) | 0.899 (0.858-0.928) | 0.871 (0.806-0.913) | 0.925 (0.894-0.947) |
| JLCA2 | 0.928 (0.899-0.949) | 0.879 (0.831-0.914) | 0.897 (0.825-0.936) | 0.927 (0.898-0.949) |
| KJLO1 | 0.966 (0.952-0.976) | 0.948 (0.926-0.963) | 0.930 (0.902-0.951) | 0.964 (0.949-0.975) |
| KJLO2 | 0.973 (0.962-0.981) | 0.969 (0.956-0.978) | 0.980 (0.970-0.986) | 0.983 (0.976-0.988) |
| WBL*KJLt1 | 0.948 (0.926-0.963) | 0.916 (0.882-0.941) | 0.890 (0.847-0.922) | 0.943 (0.919-0.960) |
| WBL*KJLt2 | 0.950 (0.929-0.965) | 0.947 (0.925-0.963) | 0.965 (0.950-0.975) | 0.968 (0.955-0.978) |

^a^SS-A and SS-B, senior specialist annotated twice; SS-M, mean of SS-A and SS-B; JR-A and JR-B, the junior resident annotated twice; AI, the deep-learning model’s prediction; ICC, intra-class correlation coefficient; CI, confidence interval; HKAA, hip-knee-ankle angle; mLDFA, mechanical lateral-distal-femoral angle; mLPFA, mechanical lateral-proximal-femoral angle; mLDTA, mechanical lateral-distal-tibial angle; WBLR, weight-bearing line ratio; FMA, femoral mechanical axis; TMA, tibial mechanical axis; mMPTA, mechanical medial-proximal-tibial angle; JLCA, joint line convergence angle; KJLO, knee joint line orientation; AJLO, ankle joint line orientation; WBL, weight-bearing line; KJLt, knee joint line of tibial side; WBL*KJLt, the angle between the WBL and KJLt.

Table 2S The error between predictions and annotations on the testing dataset^a^

| Variables | | Error (mm)  Mean±SD (95%CI) |
| --- | --- | --- |
| ROI | Hip joint | 5.0±2.1 (4.4~5.5) |
|  | Knee joint | 4.6±1.6 (4.2~5.0) |
|  | Ankle joint | 3.6±1.7 (3.1~4.0) |
| Anatomical landmarks | HJC | 1.2±0.8 (1.0~1.4) |
|  | TGR | 1.2±0.6 (1.0~1.3) |
|  | MPTP | 0.7±0.4 (0.6~0.9) |
|  | LPTP | 0.8±0.5 (0.7~0.9) |
|  | MLPTP | 1.0±0.5 (0.9~1.1) |
|  | LLPTP | 1.3±0.6 (1.1~1.4) |
|  | MLPFC | 1.2±0.7 (1.0~1.3) |
|  | LLPFC | 1.2±0.7 (1.0~1.3) |
|  | MPTD | 0.8±1.5 (0.4~1.1) |
|  | LPTD | 0.9±1.5 (0.5~1.3) |

^a^CI, confidence interval; SD, standard deviation; ROI, region of interest; HJC, hip joint center; TGR, tip of the greater trochanter; MPTP and LPTP, medial and lateral points of the tibial plateau; MLPTP and LLPTP, medial and lateral lowest points of the tibial plateau; MLPFC and LLPFC, medial and lateral lowest points of the femoral condyles; MPTD and LPTD, medial and lateral points of the talar dome.

Table 3S The average errors between the AI, JR and SS-M^a^

| Anatomical parameters | Testing dataset  (n = 122) | | | External validation dataset  (n = 150) | | |
| --- | --- | --- | --- | --- | --- | --- |
|  | JR - SS-M  Mean±SD | AI - SS-M  Mean±SD | *p* value | JR - SS-M  Mean±SD | AI - SS-M  Mean±SD | *p* value |
| HKAA, ° | -0.00±0.27 | -0.02±0.20 | 0.273 | -0.00±0.21 | -0.01±0.09 | 0.528 |
| mLDFA, ° | -0.33±0.82 | -0.04±0.56 | <0.001 | -0.12±0.66 | -0.08±0.47 | 0.502 |
| mLPFA, ° | 0.35±1.56 | 0.53±1.40 | 0.130 | 0.13±1.43 | 0.08±0.88 | 0.742 |
| mLDTA, ° | -0.01±0.88 | 0.04±1.24 | 0.698 | -0.08±0.47 | 0.10±0.70 | 0.014 |
| WBLR, % | -0.65±1.32 | 0.07±1.02 | <0.001 | -0.66±1.10 | 0.03±0.57 | <0.001 |
| AJLO, ° | -0.04±0.76 | -0.02±1.20 | 0.874 | -0.02±0.45 | -0.12±0.70 | 0.153 |
| FMA/TMA | 0.00±0.01 | -0.00±0.01 | 0.848 | 0.00±0.00 | -0.00±0.00 | 0.001 |
| mMPTA1, ° | -0.08±1.10 | -0.11±0.78 | 0.095 | -0.11±1.04 | -0.22±0.62 | 0.263 |
| mMPTA2, ° | 0.05±0.55 | -0.04±0.47 | 0.727 | 0.12±0.56 | -0.05±0.31 | 0.001 |
| *p* value | 0.837 | 0.057 |  | 0.017 | 0.003 |  |
| JLCA1, ° | 0.41±1.22 | -0.13±0.98 | <0.001 | 0.01±1.22 | -0.13±0.76 | 0.236 |
| JLCA2, ° | 0.39±0.90 | 0.02±0.78 | <0.001 | 0.24±0.85 | 0.04±0.54 | 0.017 |
| *p* value | 0.834 | 0.059 |  | 0.017 | 0.003 |  |
| KJLO1, ° | -0.11±1.09 | 0.14±0.77 | 0.025 | 0.02±1.03 | 0.20±0.63 | 0.049 |
| KJLO2, ° | -0.14±0.54 | -0.05±0.49 | 0.094 | -0.21±0.61 | 0.03±0.32 | <0.001 |
| *p* value | 0.831 | 0.015 |  | 0.017 | 0.003 |  |
| WBL*KJLt1, ° | -0.12±1.10 | 0.10±0.80 | 0.055 | 0.05±1.03 | 0.20±0.63 | 0.095 |
| WBL*KJLt2, ° | -0.09±0.54 | -0.05±0.51 | 0.385 | -0.18±0.61 | 0.04±0.33 | <0.001 |
| *p* value | 0.831 | 0.057 |  | 0.017 | 0.004 |  |

^a^AI, the deep-learning model’s prediction; JR, the junior resident’s annotation; SS-M, the mean of senior specialist’s annotation; SD, standard deviation; HKAA, hip-knee-ankle angle; mLDFA, mechanical lateral-distal-femoral angle; mLPFA, mechanical lateral-proximal-femoral angle; mLDTA, mechanical lateral-distal-tibial angle; WBLR, weight-bearing line ratio; FMA, femoral mechanical axis; TMA, tibial mechanical axis; mMPTA, mechanical medial-proximal-tibial angle; JLCA, joint line convergence angle; KJLO, knee joint line orientation; AJLO, ankle joint line orientation; WBL, weight-bearing line; KJLt, knee joint line of tibial side; WBL*KJLt, the angle between the WBL and KJLt.

Table 4S Comparison between the results on HKAA in previous studies and in ours^a^

| Author | Dataset Type  (TD or EVD) | AI algorithm | Average errors of HKAA  AI vs Ground truth | |
| --- | --- | --- | --- | --- |
|  |  |  | Mean (1.96 SD, °) | Error >1.5° |
| Pei (1) | TD | U-Net | -0.4905 (-1.9698~0.9887) | 10.83% |
| Tack (2) | TD | YARLA | 0.09 (-1.34~1.53) | 1.82% |
| Nguyen (3) | Unclear | CNN | -0.402 (-1.736~0.932) | 17.7% |
| Shock (4) | Both | U-Net | TD: 0.01 (-0.90~0.93)  EVD: 0.13 (-2.63~2.89) | - |
| Ours | Both | YOLOv5 | TD: -0.02 (-0.42~0.37)  EVD: -0.01 (-0.19~0.16) | 0% |

^a^TD, testing dataset; EVD, external validation dataset; AI, artificial intelligence; HKAA, hip-knee-ankle angle; SD, standard deviation; CNN, convolutional neural network; YARLA, YOLOv4 and Resnet landmark regression algorithm.

Table 5S Comparison between the results in Simon’s study and in ours on the external validation dataset^a^

| Author | Absolute errors  Mean (95% CI, °) | | | | | Failure rate (Error >2.0°, %) | | | | | | | | | | |
| --- | --- | --- | --- | --- | --- | --- | --- | --- | --- | --- | --- | --- | --- | --- | --- | --- |
|  | HKAA | | mLDFA | | mMPTA | HKAA | | mLDFA | | mMPTA | | JLCA | | mLPFA | | mLDTA |
| Simon (5) | 0.39 (0.35~0.44) | 0.96 (0.76~1.18) | | 1.07 (0.95~1.22) | | 1.4 | 9.0 | | 14.5 | | 20.1 | | 39.8 | | 14.9 | |
| Ours | 0.07 (0.06~0.08) | 0.33 (0.28~0.39) | | 0.45 (0.37~0.53) | | 0 | 0.7 | | 2.0 | | 2.7 | | 4.7 | | 0 | |

^a^CI, confidence interval; HKAA, hip-knee-ankle angle; mLDFA, mechanical lateral distal femoral angle; mMPTA, mechanical medial proximal tibial angle; JLCA, joint line convergence angle; mLPFA, mechanical lateral proximal femoral angle; mLDTA, mechanical lateral distal tibial angle.

1. **Backgound**

**Deep-learning Based Detection Algorithm**

In the early development stage of deep-learning detectors, networks usually include two stages: the region proposal and regression stages. YOLO (you only look once), proposed by Redmon et al., directly regresses bounding box coordinates from previously set anchors using an end-to-end network.(6) In addition, the YOLO algorithm, a representative of the single-stage network, achieves faster speed while reducing the accuracy, especially in the localization of crowded or small objects. By introducing batch-normalization techniques, intersection over union (IOU) loss, multi-scale training, and utilizing Darknet53 as the backbone, YOLOv3 achieved state-of-the-art accuracy in 2018 and faster speed. The YOLO algorithm aims to reach an optimal trade-off between accuracy and speed and is widely deployed in industrial fields. In addition, the YOLO algorithm is updated with the development of data augmentations, network designs, loss function designs, and even anchor initiation methods. Compared with YOLOv3, the advantages of YOLOv5 are listed as follows:

**3.1 Data Augmentation**

The YOLOv5 detection algorithm adopts data augmentation, including color jittering, random translation, random flip, random scaling, and mosaic. Mosaic augmentation, proposed by YOLOv5, concatenates four images into one and is beneficial for detecting small objects.

**3.2 Network design**

The networks of detection algorithms can be divided into three parts: backbone, neck, and head. The backbone of YOLOv5 is based on the Cross-Stage-Partial (CSP) network.(7) CSP network proposes cross-stage shortcuts that strengthen the learning ability of convolutional neural network (CNN). YOLOv5 uses a combination of a feature pyramid network (FPN)(8) and a path-aggregation network (PAN)(9), which aggregates features of different levels first from top to bottom and then from bottom to top. Proper design of the backbone and neck speed up training efficiency, reduces computation costs, and improves detection accuracy.

**3.3 Loss Function**

IOU loss measuring overlap situations, which are used for object localization, ignore the distance between the centers of predictions and ground truths. It is replaced with complete IOU (CIOU) loss in YOLOV5, penalizing center point and length-width errors, thus leading to better localization accuracy.

**3.4 Adaptive Anchor Initiation**

For the YOLO algorithm, the initiation of anchors should be changed because of the different datasets. The YOLOv3 algorithm uses a clustering algorithm before the training time to find the best initiation of the anchors. However, to learn the parameter settings of anchor initiation during the training period, the YOLOv5 algorithm adaptively updates the anchors using a genetic algorithm to find the best anchors in the supplied dataset.

1. **Methods**

**Hyperparameters for the deep-learning-based detection algorithm**

**4.1 Optimizer and scheduler**

final learning rate: 0.01

initial learning rate: 0.1

momentum: 0.937

weight decay: 0.0005

warmup epochs: 3.0

warmup momentum: 0.8

warmup bias learning rate: 0.1

**4.2 Loss weights**

bounding box loss: 0.05

classification loss: 0.5

objectness loss: 1.0

**4.3 Non-maximus-suppression hyperparamters**

iou_t: 0.2

anchor_t: 4.0

**4.4 Data augmentation settings**

Parameters for color jittering: hsv_h: 0.0, hsv_s: 0.7, and hsv_v: 0.4

random translate ratio: 0.1

random scaling ratio: 0.5

random flip probability: 0.5

mosaic probability: 1.0

1. **Results**

The confusion matrix of the 10 landmark classes below shows a good classification performance. The horizontal axis indicates the labeled class of a single landmark, and the vertical axis represents the predicted class from the detection model. Because all the foreground elements in the diagonal line, indicating correct detection, have values >0.9, we can conclude that the model classifies different landmarks well. Specifically, the model achieved an excellent performance in detecting landmarks, including TGR, MPTP, LPTP, MPTD, and LPTD. However, the major difficulty is confusion between the MLPTP and MLPFC and between the LLPTP and LLPFC, which occurs under ratios <0.1, which could be improved in further development.

**
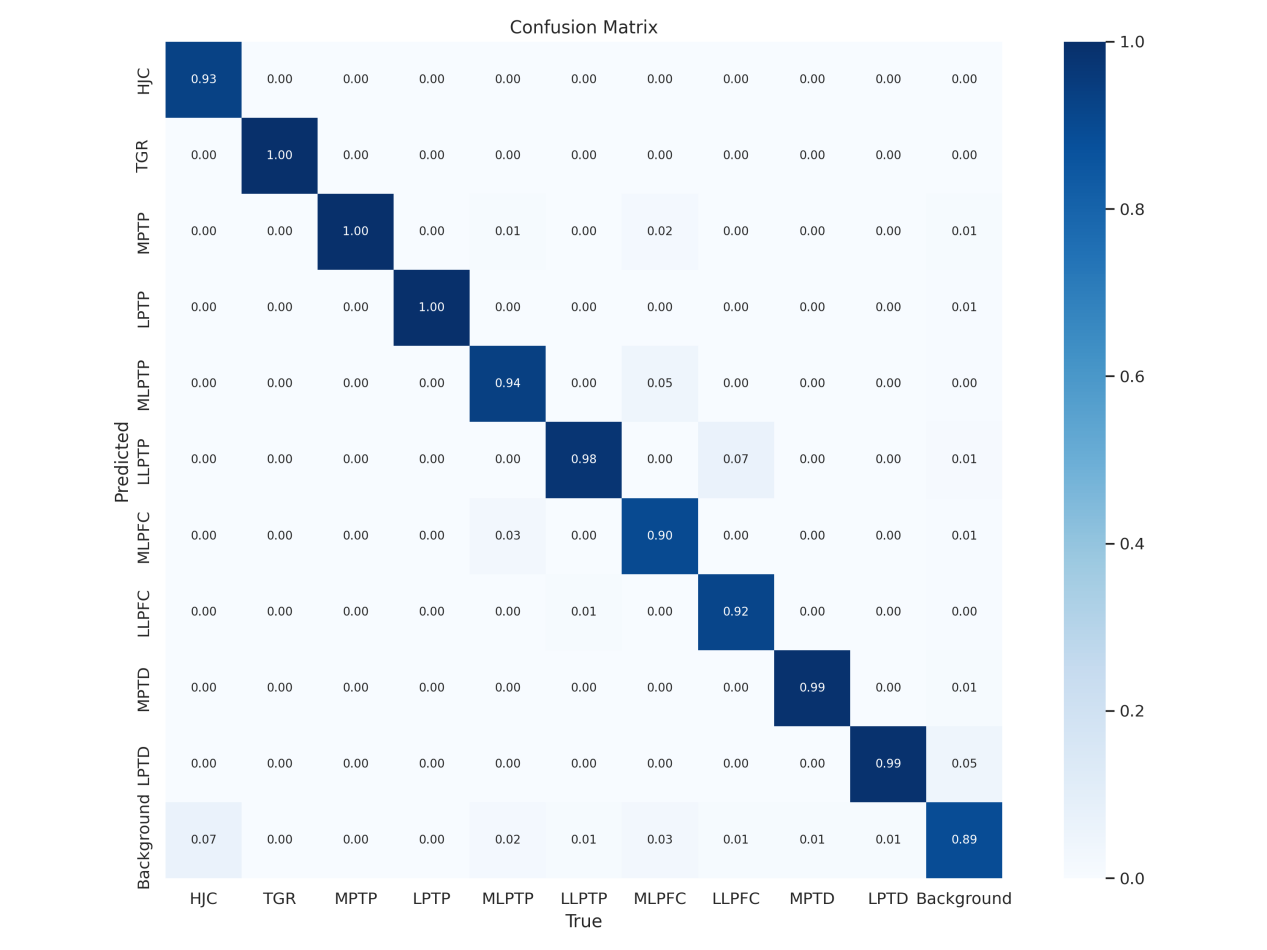
**

**Figure 1: Confusion matrix of 10 landmark classes. HJC, hip joint center. TGR, tip of the greater trochanter.** MPTP and LPTP, medial and lateral points of the tibial plateau. MLPTP and LLPTP, medial and lateral lowest points of the tibial plateau. MLPFC and LLPFC, medial and lateral lowest points of the femoral condyles. And MPTD and LPTD, medial and lateral points of the talar dome.

The precision-recall curve below shows the excellent final detection results of the 10 landmark classes. When the test-time IOU threshold was set to 0.5, the mean average precision(mAP) results of all classes were 0.990, and each class had a mAP >0.95.


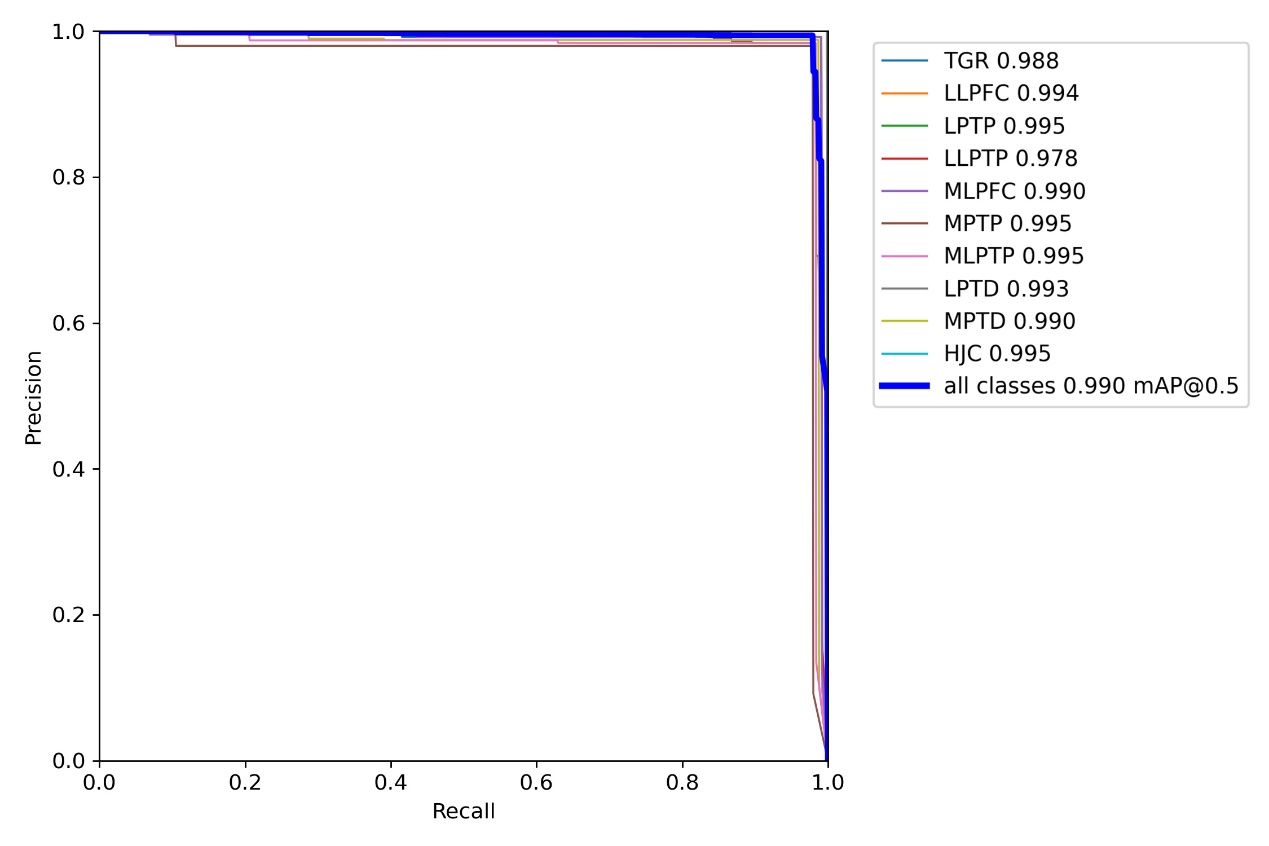


**Figure 2: Precision-recall curve. HJC, hip joint center. TGR, tip of the greater trochanter. MPTP and LPTP, medial and lateral points of the tibial plateau.** MLPTP and LLPTP, medial and lateral lowest points of the tibial plateau. MLPFC and LLPFC, medial and lateral lowest points of the femoral condyles. And MPTD and LPTD, medial and lateral points of the talar dome.

1. **References**

1. Pei Y, Yang W, Wei S, Cai R, Li J, Guo S, et al. Automated measurement of hip-knee-ankle angle on the unilateral lower limb X-rays using deep learning. Phys Eng Sci Med 2021;44:53-62.

2. Tack A, Preim B, Zachow S. Fully automated assessment of knee alignment from full-leg X-rays employing a "YOLOv4 and resnet landmark regression algorithm" (YARLA): Data from the Osteoarthritis Initiative. Comput Methods Programs Biomed 2021;205:106080.

3. Nguyen TP, Chae D-S, Park S-J, Kang K-Y, Lee W-S, Yoon J. Intelligent analysis of coronal alignment in lower limbs based on radiographic image with convolutional neural network. Comput Biol Med 2020;120:103732.

4. Schock J, Truhn D, Abrar DB, Merhof D, Conrad S, Post M, et al. Automated analysis of alignment in long-leg radiographs by using a fully automated support system based on artificial intelligence. Radiol Artif Intell 2020;3:e200198.

5. Simon S, Schwarz GM, Aichmair A, Frank BJH, Hummer A, DiFranco MD, et al. Fully automated deep learning for knee alignment assessment in lower extremity radiographs: a cross-sectional diagnostic study. Skeletal Radiol 2022;51:1249-1259.

6. Redmon J, Divvala S, Girshick R, Farhadi A. You Only Look Once: Unified, Real-Time Object Detection. IEEE Conference on Computer Vision and Pattern Recognition (CVPR) 2016,779-788.

7. Wang C-Y, Liao H-YM, Wu Y-H, Chen P-Y, Hsieh J-W, Yeh IH, et al. CSPNet: A new backbone that can enhance learning capability of CNN. IEEE/CVF Conference on Computer Vision and Pattern Recognition (CVPR) 2020,1571-1580.

8. Lin T-Y, Dollar P, Girshick R, He K, Hariharan B, Belongie S, et al. Feature Pyramid Networks for Object Detection. 30th IEEE/CVF Conference on Computer Vision and Pattern Recognition (CVPR) 2017,936-944.

9. Liu S, Qi L, Qin H, Shi J, Jia J, Ieee. Path aggregation network for instance segmentation. 31st IEEE/CVF Conference on Computer Vision and Pattern Recognition (CVPR) 2018,8759-8768.
